# Role of neurotoxicants in the pathogenesis of Alzheimer’s disease: a mechanistic insight

**DOI:** 10.1080/07853890.2021.1966088

**Published:** 2021-08-25

**Authors:** Fatema Yasmin Nisa, Md. Atiar Rahman, Md. Amjad Hossen, Mohammad Forhad Khan, Md. Asif Nadim Khan, Mumtahina Majid, Farjana Sultana, Md. Areeful Haque

**Affiliations:** a Department of Biochemistry and Molecular Biology, Faculty of Biological Sciences, University of Chittagong, Chittagong, Bangladesh; b Department of Pharmacy, International Islamic University Chittagong, Chittagong, Bangladesh; c Faculty of Pharmacy, Universiti Kebangsaan Malaysia, Kuala Lumpur, Malaysia

**Keywords:** Neurotoxicants, Alzheimer’s disease, oxidative stress, neurodegeneration, toxic metals

## Abstract

Alzheimer’s disease (AD) is the most conspicuous chronic neurodegenerative syndrome, which has become a significant challenge for the global healthcare system. Multiple studies have corroborated a clear association of neurotoxicants with AD pathogenicity, such as Amyloid beta (Aβ) proteins and neurofibrillary tangles (NFTs), signalling pathway modifications, cellular stress, cognitive dysfunctions, neuronal apoptosis, neuroinflammation, epigenetic modification, and so on. This review, therefore, aimed to address several essential mechanisms and signalling cascades, including Wnt (wingless and int.) signalling pathway, autophagy, mammalian target of rapamycin (mTOR), protein kinase C (PKC) signalling cascades, cellular redox status, energy metabolism, glutamatergic neurotransmissions, immune cell stimulations (e.g. microglia, astrocytes) as well as an amyloid precursor protein (APP), presenilin-1 (PSEN1), presenilin-2 (PSEN2) and other AD-related gene expressions that have been pretentious and modulated by the various neurotoxicants. This review concluded that neurotoxicants play a momentous role in developing AD through modulating various signalling cascades. Nevertheless, comprehension of this risk agent-induced neurotoxicity is far too little. More in-depth epidemiological and systematic investigations are needed to understand the potential mechanisms better to address these neurotoxicants and improve approaches to their risk exposure that aid in AD pathogenesis.

**Key messages**
- Inevitable cascade mechanisms of how Alzheimer’s Disease-related (AD-related) gene expressions are modulated by neurotoxicants have been discussed.
- Involvement of the neurotoxicants-induced pathways caused an extended risk of AD is explicited.
- Integration of cell culture, animals and population-based analysis on the clinical severity of AD is addressed.

## Introduction

Alzheimer’s disease (AD), the most prevalent type of senile dementia, is represented by the extracellular repository of amyloid-beta plaques and intracellular accretion of neurofibrillary tangles in the brain, causing memory, intelligence, personality and other disorders that eventually lead to death from traumatic brain injury [1]. According to the World Alzheimer’s Report 2018, a minimum of 50 million people worldwide is presumed to live with Alzheimer’s or other types of dementia. Someone in the world is developing dementia every three seconds. In the US, over 5.8 million people of all ages have Alzheimer’s, which is the sixth leading cause of death. By 2050, it is estimated that approximately 14 million American people older than 65and 106 million people globally would be dealing with the disease [2]. Alzheimer’s disease is divided into two cases, familial AD (FAD) and sporadic AD (SAD). The FAD is associated with mutations in three major genes: APP, PSEN1 and PSEN2, located at chromosomes 21, 14 and 1, respectively. On the other hand, ageing and inherited polymorphic APOE4 allele (chromosome number 19) are the major risk factors for SAD, giving rise to increased Aβ accumulation, limited Aβ disposal, higher processing of amyloidogenic APP (Amyloid Precursor Protein) and synaptic dysfunction. However, neither ageing nor APOE4 is entirely sufficient to promote the sporadic case of AD. The interface of genetic and environmental factors is thought to be involved in the development of sporadic AD [3,4].

The exact rationale behind impaired APP metabolism and deposition of amyloid plaques and neurofibrillary tangles are still vague, but many neuritic factors such as synaptic failure, cellular oxidative stress, metal ion imbalance, compromised function of mitochondria and ER, impaired neurotransmission, apoptosis along with multiple cell signalling cascades might be involved in this regard [3,5]. Numerous research experiments have shed light on the noxious effects of major neurotoxicants, including heavy metals, insecticides/pesticides, industrial/commercial pollutants, antimicrobials and air pollutants, explicitly related to the pathological development of AD. Neurotoxicants are associated with enhanced amyloid-β (Aβ) peptide formation and tau (*τ*) hyperphosphorylation, causing neuronal death. Multiple studies demonstrated that neurotoxicants elevate the Aβ_1–42_ level and stimulate the expressions of APP, BACE1 (β-site APP cleaving enzyme1), PSEN1 genes, which are implicated in Aβ production [6]. The neurotoxicants, such as heavy metals and other chemicals, also generate cellular stress, impair antioxidant function, increase the level of reactive oxygen species and change the redox status of the cells that have negative impacts on the biomolecules of the vital organs, including the central nervous system (CNS) [7]. Consequently, these mechanisms are closely associated with altered metabolism of neurons, behavioural impairments, perturbed calcium signalling, damaged mitochondrial functionality, interference with glutamate receptors and accumulation of abnormal proteins [3]. Excessive microglial activation, causing the uncontrolled production of pro-inflammatory cytokines, is mediated by neurotoxicants and associated with impaired immune functions and neuroinflammation, a causative factor of AD [8].

Considering all these phenomena, the review has focussed on the mechanisms through which various neurotoxic agents accelerate AD pathogenesis through their harmful effects at the cellular and molecular levels by modulating different biochemical processes and signalling pathways. Finally, an integration of cell culture, animals and population-based analysis on the clinical severity of Alzheimer’s disease has been investigated in this review to support the aspects of neurotoxic effects attributed to hazardous environmental agents in Alzheimer’s disease pathogenicity.

## Materials and methods

A structured literature search was conducted using numerous databases, including Google Scholar, PubMed, Medline, ScienceDirect and SpringerLink, to determine the impact of neurotoxicants on Alzheimer’s Disease. The selected articles were assessed in line with their applicability and appropriateness. The following keywords were used to conduct the literature search such as, “neurotoxic mechanism of amyloid plaque and tauopathy in AD”, “environmental toxic agents in AD pathogenicity”, “modulation of biochemical and molecular signaling cascades linked to AD by heavy metals, industrial toxic chemicals and environmental pollutants”, “epigenetic alterations in AD-linked genes relevant to toxic substance-induced neurotoxicity,” etc. According to the relevance to this review, distinction and consistency, recently published articles were selected. However, due to limited data found in recent literature (2005–2021), some old literature (1990–2004) were taken into account based on their quality of knowledge and clarity of information.

### Toxic Aβ peptide: prime neurotoxicant leads to Alzheimer’s disease

**Figure 1.**
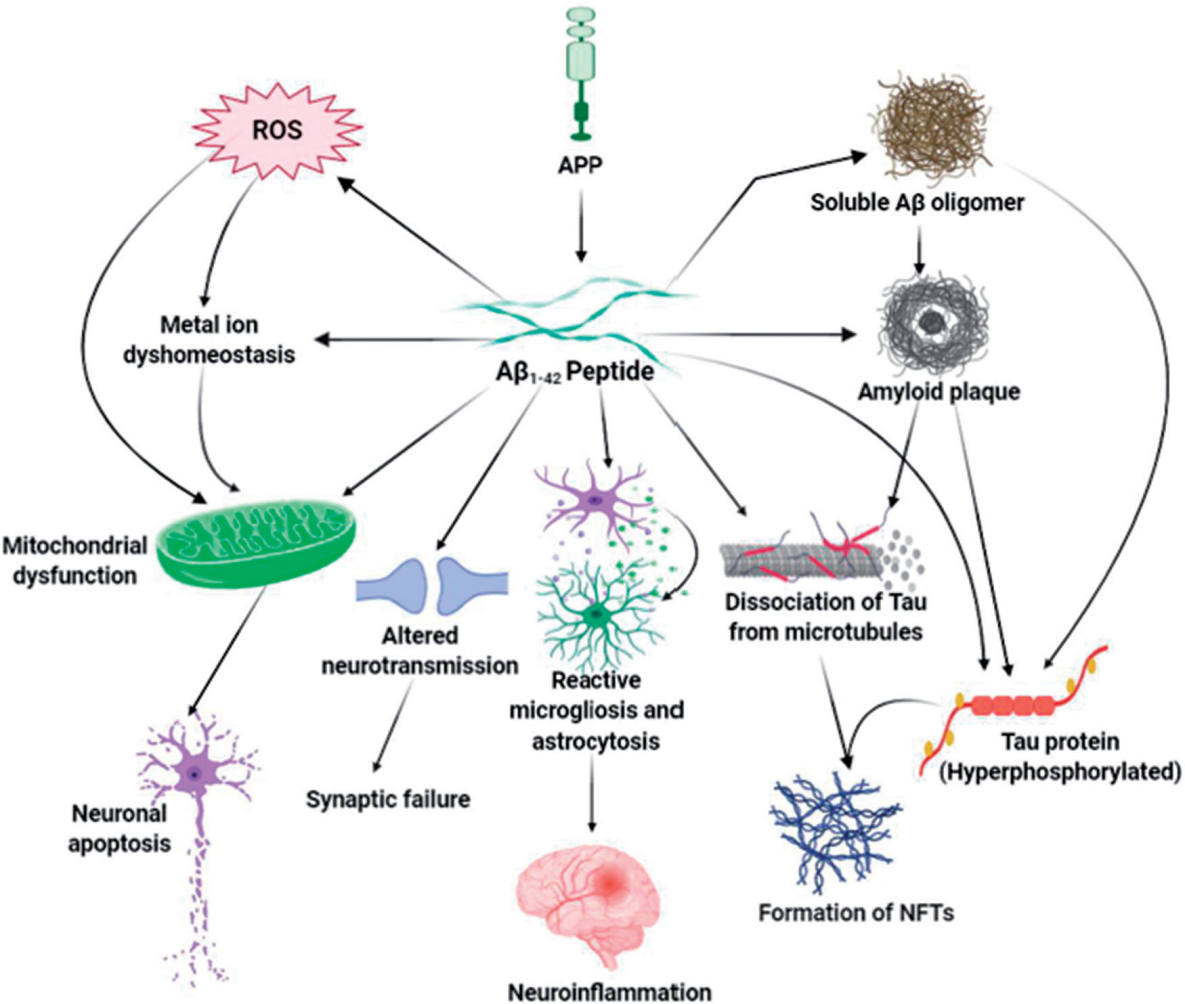
Aβ-mediated neurotoxicity in Alzheimer’s disease. The major neurotoxic effects of Aβ_1-42_ peptides in the pathological development of AD are illustrated in this diagram. That includes the formation of soluble Aβ oligomers, amyloid plaques and neurofibrillary tangles, generation of reactive oxygen species, the imbalanced concentration of metal ions, and functional impairment of mitochondria, brain immune cells, and neurotransmission. Through these mechanisms, toxic Aβ induces oxidative stress, death of neurons, synaptic malfunction, neuroinflammation, and exacerbates the abnormal protein aggregation in critical brain regions.

Neurotoxicity occurs when harmful chemicals or toxic agents affect the brain. The neurons in the adult nervous system are post-mitotic, so it is difficult to regenerate them after being destroyed. This post-mitotic nature of the neurons is the source of progressive neurological disorders, including Alzheimer's disease, which is marked by the development of senile plaques and neurofibrillary tangles, causing a significant amount of neuronal loss and disturbance of synaptic activity in the brain regions including, the hippocampus and neocortex, cortical regions that play a significant role in memory and cognitive functions [9,10]. The critical component of the senile plaque is the 42 amino acid peptide, Aβ (1-42) peptide [11]. In the amyloidogenic pathway, cleavage of APP is mediated by BACE1 and γ-secretase to generate the neurotoxic form of Aβ peptide [10]. The deposition of this toxic Aβ peptide is the prime neuropathological factor in Alzheimer’s disease [10,11]. Extensive research has emphasized that the build-up of Aβ plaques in brain regions related to memory and cognition seeds AD progression. High levels of Aβ not only destroy neurons but also impair the structure and function of microglia, astrocytes, and vascular endothelial cells of the brain. The soluble oligomeric Aβ and Aβ-derived diffusion ligands (ADDLs) or protofibrils have been suggested as the powerful toxic form of amyloid plaques [9]. Aβ targets many cellular processes, including oxidative stress, inflammation, mitochondrial function, membrane permeability, excitotoxicity and synaptic neurotransmission to promote neurodegeneration (Figure 1) [12].

#### Aggregates of hyperphosphorylated tau protein: a primary marker for AD

The neurotoxic function of Aβ is intrinsically linked to tau (*τ*), a microtubule-associated protein that provides structural assembly and stability of cytoskeletons [10,11]. Tauopathy is defined as the neurotoxic effects of pathological NFT formation caused by an aberrantly folded form of hyperphosphorylated tau, and it has been linked to AD and other neurodegenerative diseases. Internalization of Aβ inside the cells potentially stimulates NFT generation by Aβ; wherein perturbation in tau hyperphosphorylation and NFT formation negatively impacts Aβ aggregation and plaque composition [9,10]. Aβ-mediated tau pathology is stimulated by activation of tau kinases that facilitate the hyperphosphorylation of tau, failure in the proteasomal function, which reduces tau degradation, and the initiation of the caspase-3 activity; ultimately, causing an irregular tau aggregation [13]. The expression of tau is critical during Aβ-mediated synaptotoxic processes where Aβ peptides target phosphorylation-based pathways. In these signalling pathways, tau appears either as a signal transducer or an effector molecule [11]. Tau is hyperphosphorylated by GSK-3β (glycogen synthase kinase 3 beta) and other kinases regulated by both Aβ peptides and APOE4, and this kinase-mediated hyperphosphorylation of tau contributes to the dissociation of tau from microtubules. Aβ oligomers assemble tau proteins; after removing them from microtubules, alter the structure of neurites giving rise to synaptic malfunction and neuronal death [9].

#### Oxidative stress, metal ion dyshomeostasis and mitochondrial dysfunction

Multiple cellular stress markers, including reactive oxygen species (ROS), reactive protein carbonyls, 8-hydroxyguanine and lipid peroxides, are prominent in AD brains [11]. In the case of AD, Aβ is both the cause and result of oxidative stress. The Methionine 35 residue of Aβ _1–42_ is essential for oxidative stress induction and neurotoxicity of Aβ peptides [3]. Aβ removes protons from adjacent proteins and lipids through its radical form and produces carbonyls and lipid peroxides [14]. At synapses, Aβ elicits the production of cytotoxic aldehyde-4-hydroxynonenal (4-HNE) and lipid peroxides that enhance the susceptibility to neuronal apoptosis. During Aβ accumulation, hydrogen peroxides (H_2_O_2_) are produced immediately. Amendment in the oxidative condition of the brain sets off the production and deposition of Aβ through elevated β-secretase function. Disruption in metal ion homeostasis is consistent with the cellular stress observed in the AD brain [3]. Some metal-binding regions have been found in Aβ peptides where Cu^2+^, Zn^2+^, Fe^3+^ and other metal ions are shown to be inhabited in the Aβ oligomers of the amyloid plaques [9,12]. After being combined with Aβ peptide, these metals facilitate Aβ aggregation and amyloid plaque formation. Cu^2+^ and Fe^3+^ are reduced by Aβ, which is a single electron process. After that, the reaction of these reduced metals with the O_2_ molecule produces H_2_O_2_ and triggers lipid peroxidation [9,12]. Products of lipid peroxidation such as HNE (hyroxynonenal) and other responsive carbonyl components initiate apoptotic signals. The formation of HNE/Aβ adjuvant by HNE fastens the oligomerization of Aβ. Furthermore, HNE oxidatively converts tau to its aggregated form that develops NFTs [3,9]. Conjugates of Aβ and Cu^2+^ produce H_2_O_2,_ contributing to di-tyrosine cross-linked Aβ oligomers [11]. Aβ injures the blood-brain barrier (BBB) by generating superoxides. In the vasculature, Aβ-Cu conjugates yield superoxide ions and reduce blood supply to the brain by reacting with nitric oxide (NO) implicated in vascular dementia and AD [9]. In mitochondria, Aβ produces ROS and dampens the enzymatic activity of the electron transport system (ETS), resulting in mitochondrial dysfunction and impaired energy metabolism. Toxic amyloid peptides damage mitochondrial membrane permeability by forming transition pores that liberate cytochrome c and stimulate mitochondrial apoptotic death signalling [3,9]. Aβ binds with the CD36 receptor expressed in endothelial cells and microglia and generates ROS, vasoconstriction, and alters the vascular tone, leading to neuronal damage [10].

#### Altered Ca^2+^level, apoptosis, and neuroinflammation

Homeostasis of Ca^2+^ is necessary for memory and cognition while jeopardizing Ca2+ homeostasis is linked to AD-related complications. Exacerbated Aβ_1–42_ formation disrupts intraneuronal Ca^2+^ concentrations. Aβ mediated cellular stress and reactive oxidative species alter Ca^2+^ regulation and damage Ca^2+^-ATPase pumps in synaptosomes and trigger Cu^2+^ influx through ion channels and glutamate receptors associated with impaired synaptic plasticity in the hippocampus [3]. Enhanced expression and function of ryanodine receptors found in neurites are induced by Aβ peptides that perturbs neuronal Ca^2+^ homeostasis [10]. Elevated levels of intracellular Ca^2+^ stimulate calpains that induce apoptotic death signals at synaptic terminals. Aβ causes an alteration in cell cycle activities leading to neuronal death. Aβ-induced oxidative stress triggers JNK protein kinase that determines the expression of several genes essential for apoptosis. The stimulation of apoptotic caspases is connected to Amyloid plaque formation. Besides, Aβ deposits activate caspase and induce caspase-mediated cleavage of tau along with impaired cognition. The cleavage of tau facilitated by caspases enables tau as a stimulating apoptosis agent [3]. Aβ aggregates stimulate the reactive state of microglia and increase the pro-inflammatory response of microglia by NF-κB activation, which modulates extracellular signal-regulated kinase (ERK) and mitogen-activated protein kinase (MAPK) cascades and leads to the expression of numerous inflammatory receptors, cell surface markers, and cytokines. Additionally, Aβ prompts inflammatory cytokine production by TLR heterodimers, which CD36 controls. Astrocyte is activated through this pro-inflammatory stimulus and further generates cytokines, chemokines, and acute-phase proteins. Uncontrolled reactivity and expansion of microglia and astrocytes exacerbate cytokine production that causes neuroinflammation and neuronal death [3,10].

#### Impaired neurotransmission: worsening of AD

Cholinergic functions such as choline acetyltransferase (ChAT), acetylcholinesterase (AchE), and acetylcholine are defective in Alzheimer’s disease. In synaptic cleft, acetylcholine production, delivery, and degradation are influenced by Aβ peptides. Aβ elevates the function of AChE near amyloid plaques and NFTs. Besides, the AChE enzyme enhances Aβ deposition. Association of Aβ peptides to a7 subtype of nicotinic acetylcholine receptors (a7nAChR) causes the acceleration of the N-methyl-D-aspartate receptor (NMDAR) endocytosis and alteration in nicotinic and MAPK signalling resulting in the impairment of cholinergic neurotransmission and cognitive deficits in AD [3,10]. Aβ peptides interfere with glutamate receptors and transporters that adversely bring changes in glutamatergic neurons. Aβ causes neuronal susceptibility to glutamate excitotoxicity. Interaction of Aβ with N-methyl-D-aspartate (NMDA) and α-amino-3-hydroxy-5-methyl-4-isoxazolepropionic acid (AMPA) receptors leads to glutamate dyshomeostasis, excitotoxicity, and defective plasticity [10]. The function of a tyrosine-protein kinase, Fyn, is necessary for Aβ toxicity. It phosphorylates NMDARs and alters synaptic transmission in cooperation with modified transport and activities of NMDARs mediated by Aβ [11]. Oligomeric Aβ fluctuates glutamatergic neurotransmission and is responsible for synaptic loss [15]. Aβ prevents long-term potentiation (LTP) and augments long-term depression (LTD) due to deviation in glutamate reabsorption [12]. Increased Aβ oligomers incompletely inhibit NMDAR and generate LTD that causes shrinkage of neurites and synaptic injury. Soluble Aβ oligomers impede glutamate absorption at synapses and enhance their concentration in the synaptic cleft. As a result, synaptic NMDARs are activated and then become desensitized, facilitating synaptic depression. Also, Aβ mediated glutamate spill-over stimulates extrasynaptic NMDARs and mGluRs that critically induce LTD and inhibit LTP [15]. Exaggerated stimulation of extrasynaptic NMDARs is interlinked with the toxicity mediated by Aβ peptides [3].

### Major neurotoxicants in the pathogenesis of AD

**Figure 2.**
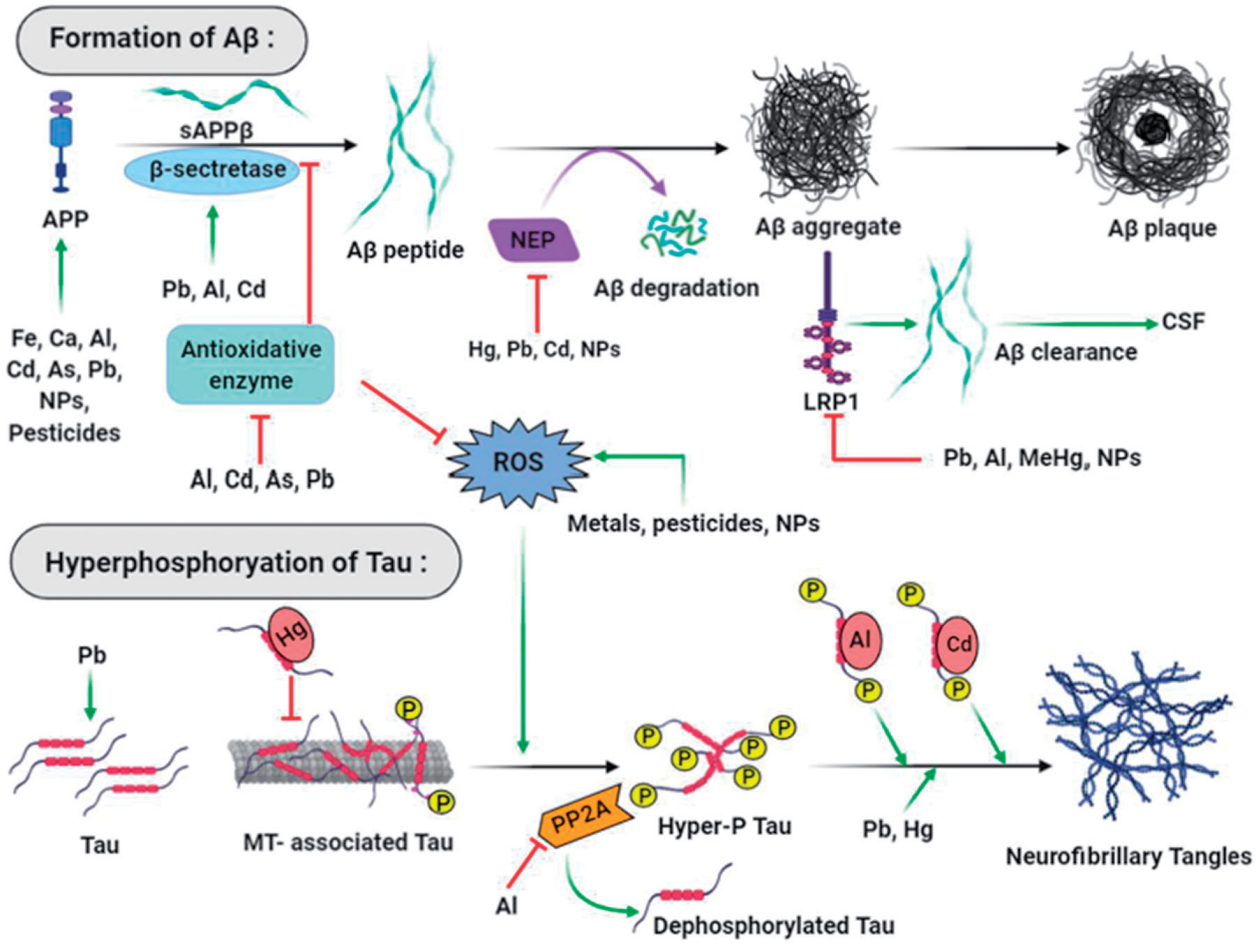
Major neurotoxicants at the onset and progression of Alzheimer’s disease. The abnormal build-up of amyloid-beta protein that generates amyloid plaques and hyperphosphorylated tau protein that forms neurofibrillary tangles in and around neurons are apparent at the onset of Alzheimer’s disease. Multiple neurotoxicants such as metals, pesticides, and nanoparticles have been found to augment the formation of Aβ aggregates and NFTs through different mechanisms. These neurotoxicants produce oxidative stress in neurons that trigger Aβ peptide formation and hyperphosphorylate tau protein. Neurotoxicants stabilise APP expression and β- secretase enzyme activities; on the other hand, they disrupt the functions of antioxidant enzymes, Aβ degrading proteins, and receptors that result in amyloid plaque formation. Neurotoxicants bind with tau, dissociate them from microtubules and increase their hyperphosphorylation. Enzymes that dephosphorylate tau protein are also inhibited by neurotoxicants that ultimately leads to the formation of neurofibrillary tangles.

Risk exposure to heavy metals, toxic industrial chemicals and air pollutants is widely highlighted in the onset and progression of AD; however, it is still vague if a single factor or a combination of these precarious elements facilitates the neurodegeneration process (Figure 2) . The BBB is exclusively significant for the maintenance of neuronal microenvironment and CNS homeostasis. Optimum balance of essential metals such as calcium, copper, iron, magnesium, manganese and zinc, etc., are vital for numerous brain functions. The BBB vasculature contains a range of active and receptor-mediated transport systems to monitor the metals’ passage in the brain. Thus, other non-essential metals such as arsenic, cadmium, lead and mercury, etc., can cross the BBB with the help of those transporters. Transport of other chemical substances is contingent on their degree of lipophilicity [16]. Some epidemiological studies reveal that air pollutants, on long-term exposure, cause the decrease in tight junction protein-1 and increase in vascular cell adhesion molecule-1 and intracellular adhesion molecule-1, which enhance the permeability of BBB. In this article, we outlined the fundamental neurotoxic mechanisms of five categories of neurotoxicants, including 1) heavy metals; 2) pesticides/insecticides; 3) antimicrobials; 4) air pollutants; and 5) industrial/commercial chemicals, etc.

#### Heavy metals

Metal toxicity is a widespread global episode that mediates multiple pathogenic mechanisms on the human body. Metal ion imbalances are associated with numerous degenerative diseases, including cancer, diabetes, and neurological disorders like Alzheimer's and Parkinson's disease [17]. Researches have confirmed that excessive iron (Fe) deposition in the hippocampus and cerebral cortex regions is closely linked to Alzheimer’s disease. An *in vitro* research has shown that Fe with aluminium (Al) is involved in the development of Aβ 42 aggregates with β-pleated sheet conformations to form amyloid fibrils. Copper (Cu) is associated with the neurotoxic mechanisms in AD. The redox properties of Cu are essential for brain cells’ enzymatic functions and control cellular biochemical reactions [18]. The participation of Cu in redox reaction in the brain tissue generates ROS, damaging biomolecules and ultimately progresses towards AD. Oxidative damage and neuropathology of AD arise from elevated serum levels of redox-active Cu in the brain. Some studies have revealed that the binding affinity of Cu^2+^ to the N-terminal domain of APP can disrupt synaptogenic function, stability, and metabolism that dramatically enhances the pathological progression of AD [19]. Zinc (Zn) is a trace element that accumulates in the brain tissue at the highest level than other body organs. Zn is present in the brain as a free ion (Zn^2+^) and enriched within the region of the synaptic and glutamatergic nerve terminals, affecting glutamatergic and GABA (gamma-Aminobutyric acid) receptor activities. Therefore, Zn is linked to the balance of excitation and inhibition of neurotransmission, alteration to which causes loss of memory function [20,21]. Zn homeostasis is critical for many neurological disorders. An excessive Zn in the brain ECF has also been suggested to promote neuronal toxicity and protein aggregation [22]. Manganese (Mn) is vital to brain development and function. However, a high level of Mn in brain tissue can increase the development of AD pathology. A recent study has shown that MnSOD (Manganese superoxide dismutase) deficiency causes oxidative stress, loss of mitochondrial function, and raises the levels of Aß plaque in the brain [23]. Lead (Pb) is a well-known neurotoxic redox metal that causes oxidative stress that results in cholinergic dysfunction, neuroinflammation, mitochondrial damage, and neuronal apoptosis [24,25]. Pb-induced neurotoxicity inhibits the function of NMDARs and generates neuroinflammation. Excessive Pb accumulation in the blood and brain regions promotes epigenetic alteration due to latent early life exposure [26,27]. Mercury (Hg) is also considered a risk factor for Alzheimer's disease because of its neurotoxic properties. An animal model has indicated that Hg-induced neurotoxicity is responsible for mitochondrial dysfunction, oxidative stress, neuroinflammation, and apoptosis leading to cognitive decline [28]. Al is a non-essential metal associated with neurotoxicity which can cross BBB. Drinking water with high Al concentration may cause cerebral impairment and short-term memory loss [29]. Al influences more than 250 biological reactions including axonal transport, neurotransmitter synthesis, synaptic transmission, gene expression, oxidative stress and inflammatory responses, etc. However, the pathogenic mechanism of Al in the brain is still uncertain [30].

#### Pesticides/insecticides

To meet the growing demand of an ever-increasing population, synthetic pesticides are widely used in agricultural food products. Incidences of several neurodegenerative diseases like PD, AD have been linked to exposure to these pesticides. Many pesticides target the central nervous system, disrupting cell signalling and neurochemical processes that trigger adverse reactions and neurotoxicity [31]. Several studies have shown that chronic exposure to pesticides poses a higher risk of cognitive, behavioural, psychomotor, and post-life AD. Organophosphates, carbamates, and organochlorines are among the largest pesticides, mainly known as anticholinesterases (antiACHs). These pesticides elicit neurotoxicity by interfering with cholinergic functions. They phosphorylate the active site of the acetylcholinesterase enzyme and halt the breakdown of acetylcholine. Accumulation of an increased amount of acetylcholine in the brain leads to neuronal death and cognitive impairment [32,33]. Another study has depicted that, among others, organophosphates and organochlorines, extremely interminable pesticides cause oxidative stress and neurotoxicity. The exposure of these two pesticides affects glucose and lipid metabolism and the function of endocrine and excretory systems, resulting in their bioaccumulation associated with AD risk [17,34]. Rotenone, also known as fish toxin, enhances neurotoxicity by preventing mitochondrial chain function that poses a risk for AD development [35]. Paraquat (PQ) is the most commonly used organic herbicide that poses a significant risk factor for developing neurodegenerative diseases such as AD and PD. PQ exposure upregulates Aβ levels, induces oxidative stress, and impairs mitochondrial energy metabolism linked to dysfunctional memory and cognition [36]. Fipronil is a phenyl pyrazole insecticide that exerts its function through the inhibition of insect GABA receptors. Research on zebrafish embryos has shown that fipronil impairs the spinal locomotive pathways and induces neurodegeneration [37]. In the AD brain, reduction and remodelling of GABAergic neurotransmission are similar to fipronil toxicity [38].

#### Antimicrobials

Non-halogenated and polychlorinated compounds have been used as antimicrobials, preservatives, and active ingredients for pharmaceutical, dietary, and personal care. However, such classes of compounds have cytotoxic, immunotoxic, and neurotoxic effects. Several research studies have indicated that excessive use of antimicrobials and disinfectants may result in a health burden leading to AD pathogenesis [39,40]. Hexachlorophene (HCP) belongs to the aromatic compound used for multiple purposes including, disinfection and preservation, responsible for developmental neurotoxicity. Indications from some *in vivo* and *in vitro* animal models have revealed that HCP dramatically decreases succinate dehydrogenase activity, changes brain metabolic activities, and leads to vacuolar encephalopathy in the brain causing AD-like symptoms [41]. Triclocarban (TCC) and triclosan (TCS) are used as antimicrobials in skincare and personal hygiene products, particularly in bar soaps and toothpaste, to destroy and inhibit the growth of microorganisms. TCS interferes with type 1 ryanodine receptors and alters Ca^2+^ homeostasis in the brain that develops neurotoxicity, ultimately leading to neurological disorders [42]. In *utero* exposure to TCC alters behavioural development and neurogenesis that affect the survival time of female murine neonates [17]. Similarly, a study has disclosed that excessive TCC and TCS exposure on the slender freshwater fish brain leads to increased oestrogen that causes neurotoxicity [43]. The significance of TCC and TCS exposures in AD development is still unclear, and further investigation is suggested. Parabens belong to the preservatives class, which are most widely used in cosmetic and pharmaceutical products due to their antifungal and antibacterial activities. *In vivo* experiment on fish, the model has indicated that bioaccumulation of paraben in the brain after exposure, leads to decreased neurotransmitter activity and causes neurological changes in behaviours [44]. Another study has reported that swine offsprings are vulnerable to butylparaben that induces anxiety, behavioural changes, and learning-like difficulties at adult ages [39].

#### Air pollutants

The accretion of free radicals is a long-lasting mechanism that contributes to neuropathology and neuroinflammation later in life. Recent experimental studies have reported that particulate matter in the air may be responsible for developing neurological disorders, mostly PD and AD. The presence of toxic metals such as Pb, Ni, and gasses such as SO_2_, CO, and NO_2_ in contaminated air contributes to reactive oxygen species, cerebrovascular vandalization, neuroinflammation, oxidative stress, and Aβ peptide accumulation associated with AD development [45]. An experiment has shown that nickel (Ni) exposure on rat brain triggers a rise in AD-associated complications with the increase of amyloid-β42 and amyloid-β40 [46]. Moreover, two studies on young Mexican children have indicated that polluted air exposure containing particulate matter expands the piling up of proteins analogous perceived in the early stage of AD [46]. When ozone is exposed to adult Wistar rats, it generates ROS, induces neuroinflammation, and reduces brain regeneration in AD susceptible brain regions. Moreover, experimental animal and human models have revealed that the increase of organic compounds, gases, PM (particulate matter), and metals in polluted air upregulates neurological stress markers contributing to the aetiology of AD [47]. Research studies, including clinical, observational, epidemiological, and experimental (*in-vivo, in-vitro*), have demonstrated that air pollution disrupts the function of the CNS that leads to AD.

#### Industrial/commercial chemicals

Industrialization and urbanization contribute to a massive increase in pollution, contributing to serious health issues for humans and other organisms. Natural or synthetic contaminants accumulate in different parts of the human body and cause severe harmful reactions that lead to toxicity. Dioxins and dioxin-like chemicals are incredibly toxic, by-products of industrial processing of polychlorinated biphenyls (PCBs), polychlorinated dibenzo-p-dioxins (PCDDs), polychlorinated dibenzofurans (PCDFs), and polycyclic aromatic hydrocarbons (PAHs), which mediated their toxicity through aryl hydrocarbon receptors (AhRs) [48]. AhR is a transcription factor crucial for neurogenesis and memory function of the hippocampus. An experiment demonstrated that 2,3,7,8-tetrachlorodibenzo-p-dioxin (TCDD) alters AhRs expression and function, leading to neurodevelopmental dysfunction [49]. Phthalates and bisphenol-A (BPA) are widely used as plastic products and plasticizers in the food and beverage industries. Research has shown that phthalates and bisphenols can exude from prepared containers in food or beverages, resulting in epigenetic changes and other health consequences for humans over a prolonged period. Exposure to BPA in animal models has reported impaired reproductive functions and changes in animal behaviour. Besides, BPA can interfere with the formation of spine synapses in the prefrontal cortex and hippocampus, which later promotes similar clinical consequences as AD [50]. Some epidemiological studies indicated that girls are more prone to phthalate neurotoxicity than boys, while antepartum exposure to phthalates causes memory dysfunction [51]. According to a recent study, BPA interferes with insulin signalling in the brain, which is critical to extinguishing the effects of AD-related pathological proteins [52].

### Mechanism of neurotoxicants in inducing Alzheimer’s disease

#### Major signalling cascades modulated by neurotoxicants

Distinctive features of Alzheimer’s disease include extracellular Aβ aggregates, intracellular tau (*τ*) entanglements, synaptic and cholinergic malfunctions, inflammation, and substantial oxidative stress. Recently some forms of cell signalling pathways have been suggested to be involved in the pathophysiological progression of the disease [5]. Neurotoxicants aim at least one of these signalling pathways to elicit their toxic effects (Table 1). After exposure to the brain, they interact with their intended targets that lead to activation or inhibition of particular molecules, proteins, enzymes, or transcription factors and subsequently diminish the regular activities of those signalling pathways. The following section discusses some of the major signalling pathways related to Alzheimer’s and how neurotoxicants affect them.

**Table 1.** Signalling pathways modulated by neurotoxicants in the pathogenesis of Alzheimer’s disease.

Sl	Neurotoxic agents	Study type	Molecular targets	Mechanism of action	References
Wnt signalling pathway					
1	Antimony (Sb)	*In vitro*	β-catenin and GSK-3β	Activation of GSK-3β and suppression of β-catenin; reduction of Wnt/β-catenin activities	[53]
2	Arsenic (As)	*In vitro*	GSK-3β	Increased activity of GSK-3β kinase	[54]
3	Bisphenol-A	*In vitro* *In vivo*	Dkk1, GSK-3β, Wnt-1, Wnt-3, LRP-5/6, Dvl, LEF-1, TCF, β-catenin and cyclin D-1	Upregulation of Wnt signalling antagonists Dkk1 and GSK-3β;decreased level of Wnt-1, Wnt-3, LRP-5/6, Dvl, β-catenin, Wnt target gene cyclin D-1, and nuclear transcription factor LEF-1, TCF; inhibition of the Wnt/β-catenin pathway	[55]
4	Copper (Cu)	*In vitro*	GSK-3β	Increased activity of GSK-3β; phosphorylation of APP and tau	[56,57]
5	Iron (Fe)	*In vitro*	GSK-3β	Induction of oxidative stress; activation of GSK-3β kinase; hyperphosphorylation of tau	[57]
6	Methyl mercury (MeHg)	*In vitro*	GSK-3β	Higher expression of GSK-3β; suppression of neuronal proliferation and development	[58]
7	MPP+	*In vitro*	Dkk1	Induction of Dkk1; inhibition of the canonical Wnt pathway	[59]
8	Paraquat (PQ)	*In vivo*	GSK-3β	Enhanced expression and activity of GSK-3β; formation of neurofibrillary tangles (NFT); disruption in neuronal function	[60]
9	Pesticides – Deltamethrin & Carbofuran	*In vivo* *In vitro*	GSK-3β	Increased activation of GSK-3β; Tau pathology; cognitive injury	[61]
10	Silica nanoparticles (SiNPs)	*In vitro*	GSK-3β	Stimulation of GSK-3β activities; pathological deposition of Aβ; increased in tau phosphorylation	[62]
Autophagy and mTOR signalling					
1	Arsenic (As)	*In vivo*	mTOR, Beclin1, LC3, Atg, p62	Increased Beclin1, LC3, and Atg12 and decreased mTOR proteins activated autophagy; increased p62 led to autophagy dysfunction	[63]
2	Cadmium (Cd)	*In vitro*	Ca^2+^ ion and mTOR	Increased level of intracellular Ca^2+^; activation of mTOR; induction of apoptotic cell death	[64]
3	Copper (Cu)	*In vitro*	Autophagic pathway	Enhanced autophagic influx; defect in autophagic activities; neuronal cell death	[65]
4	Iron (Fe)	*In vitro*	Autophagic pathway	Increased oxidative stress concomitant with increased autophagy activation	[66]
5	Methyl mercury (MeHg)	*In vitro*	LC3II, P62 and lysosomes	Accumulation of autophagosomes; defected autophagy as well as impaired lysosomal activity; neuronal cell death	[67]
6	Manganese (Mn)	*In vivo*	Beclin1, LC3II, mTOR, and lysosome	Activation of mTOR; dysregulation in autophagy; decreased expression of beclin1 and LC3II; defected lysosome structure	[68]
		*In vitro*	Lysosome	Enhanced lysosomal permeabilization; cell death	[69]
7	Lead (Pb)	*In vivo*	Autophagic pathway	Upregulation of autophagosome formation and autophagic activity; excessive autophagy leading to cellular death	[70]
PKC signalling					
1	Aluminium (Al)	*In vitro* *In vivo*	PKC	Remarkable decrease in PKC concentration	[71]
2	Lead (Pb)	Literature review	Ca^2+^ and PKC	Interaction with PKC through Ca^2+^ substitution; activation of PKC at lower concentration	[72]
		*In vitro*	PKC	Inhibition of PKC at higher concentration	[73]
		*In vitro*	PKC	Interference with the catalytic domain of PKC that limits their function	[74]
3	Mercury (Hg)	*In vitro*	PKC	Inhibition of PKC activity	[75]
4	Methyl mercury (MeHg)	*In vitro*	PKC	Inhibition of PKC function	[76]
5	Nickel (Ni)	*In vitro*	Genes of PKCγ, PKCζ, RACK-1, and PKCδ binding protein	Reduced transcription the genes encoding PKCγ, PKCζ, RACK-1, and PKCδ binding protein	[75]

##### Inhibition of the Wnt signalling pathway by neurotoxicants

Wnts are an enormous group of 19 cysteine-rich secreted glycoproteins that bind to extracellular Frizzled (Fz) transmembrane receptors to stimulate some intracellular signal transduction cascades, including the canonical Wnt/β-catenin dependent pathway and the non-canonical β-catenin independent pathway composing of the Wnt/Ca^2+^ pathway and Wnt Planar Cell Polarity pathway, also known as the Wnt/PCP-JNK pathway [76]. The canonical Wnt/β pathway is the most comprehensively studied, which governs the Wnt target gene expressions through cytoplasmic β-catenin maintenance. After synthesis, Wnt proteins undergo different post-translational modifications in the endoplasmic reticulum (ER) followed by intracellular trafficking through various extracellular transporters. Wnt proteins serve as morphogen gradients to instigate their regulatory effects on target cells [77]. Wnt proteins bind to Frizzled (Fz) receptors and LRP5/6 co-receptors; then, the Dishevelled (Dvl) scaffold proteins and Axin are assigned in the cytoplasm triggering the assembly of β-catenin in the cytoplasm and their translocation into the nucleus. The association of β-catenin with transcription factors TCF/LEF initiates the transcription of Wnt target genes. Meanwhile, β-catenin in the cytoplasm is phosphorylated by GSK-3β due to the absence of Wnt ligands, which results in its breakdown [78].

**Figure 3.**
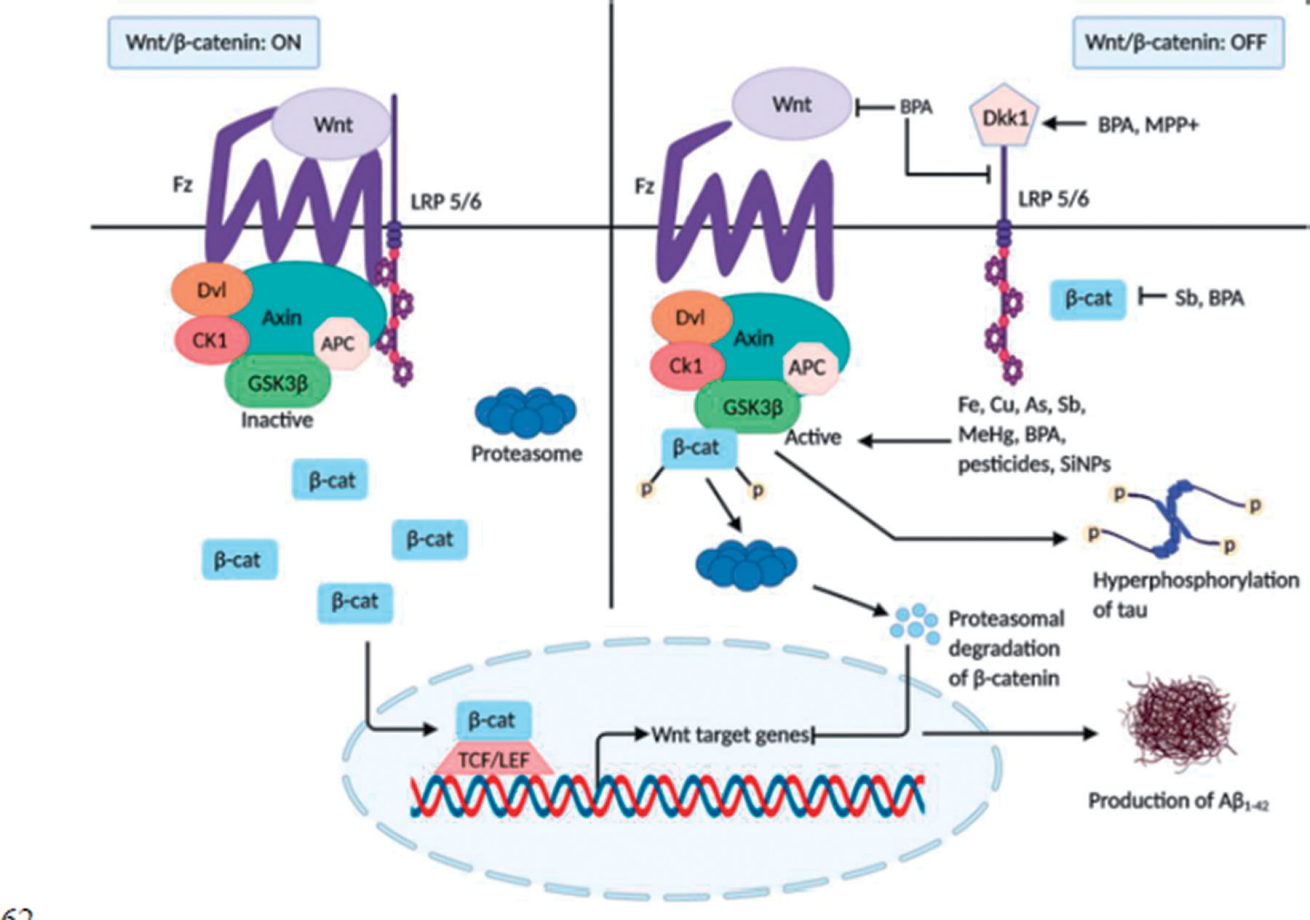
Inhibition of the Wnt/β-catenin signalling by neurotoxicants. In normal brain, when Wnt signalling is switched on, GSK-3β is found to be inactive, and tau protein remains dephosphorylated. β-catenin translocation in the nucleus activates Wnt target genes that inhibit the development of Aβ_1–42_. In the presence of various neurotoxicants (Fe, Cu, As, Pb, MeHg, BPA, pesticides, and NPs), Dkk1 and GSK-3β, the inhibitors of Wnt signalling cascade become activated. Activation of Wnt proteins, LRP 5/6, and β-catenin are also inhibited by some neurotoxicants (BPA, Sb). β-catenin is phosphorylated by GSK-3β and undergoes proteasomal degradation. As a result, Wnt signalling is shut off, leading to tau hyperphosphorylation and Aβ_1–42_ production and aggregation that aids AD pathology.

The Wnt signalling pathways play a crucial role in the central nervous system during all phases of neuronal growth and development, such as neurogenesis and synaptogenesis, and remain significant in the adult nervous system [79]. In the adult mammalian brain, matured functional neurons are generated from adult neural stem cells through a process known as adult neurogenesis, which is critical for hippocampus-dependent memory and behaviour. A growing number of studies on the neurotoxic effects of environmentally toxic substances stated that interruption in adult neurogenesis is strongly linked to neurodegeneration [80]. Multiple signalling pathways involving antioxidants, peroxisome proliferator-activated receptor (PPAR) α and γ, nicotinic and muscarinic ACh receptors, and anti-inflammatory pathways are triggered along with the Wnt signalling pathway, which contributes to its neuroprotective role against neuronal adversity. Consequently, a formidable association between AD pathology and altered Wnt signalling was proposed by several types of researches [81]. Altered function of Wnt signalling components was detected in AD brain, including downregulation of β-catenin translocation into the nucleus in a transgenic murine model, decrease in β-catenin protein levels in AD patients, elevated expression and activation of Wnt antagonist GSK-3β and Dkk-1 in post-mortal AD brain and transgenic mouse model [5,82]. Overexpression and activation of GSK-3β are related to impaired memory formation and spatial learning; also, GSK-3β participates in oxidative stress generation and tau hyperphosphorylation that disrupt neuronal function, all of which are characteristic deficits of AD [82,83]. Increased level of Dkk-1 protein is involved with age-related tau pathology, depletion of synaptic proteins and number of pre-synaptic sites causing synaptic disassembly, and increased cognitive dysfunction [5,81,82]. It is found that some neurotoxicants are associated with the disruption of these Wnt components affecting the signalling cascade and thereby leading to neurodegeneration (Figure 3).

Cu and Fe have been found to activate GSK-3β kinase activities that promote the formation of both Aβ aggregates and τ- hyperphosphorylation [56,57]. BPA, an industrial chemical used in food packaging, alters the Wnt/β-catenin signalling pathway. Both *in vivo* and *in vitro* studies on rat brains have revealed that BPA treatment substantially upregulates the Wnt antagonist genes Dkk1 and GSK-3β and reduces the level of Wnt-1, Wnt-3, LRP5/6, Dvl, and β-catenin genes. Additionally, the nuclear transcription factor (LEF-1 and TCF) and Wnt target gene expression were downregulated [55]. Pesticides such as deltamethrin and carbofuran stimulate GSK-3β activation, generating AD-related τ pathology and cognitive injuries in rats [60]. Long-term administration of paraquat (1,1′- dimethyl-4,4′-bipyridinum dichloride, PQ), a potential herbicide, enhances the level and activation of GSK-3β kinase enzyme in a rat model [60]. Another *in vitro* study on a rat model has shown that a low concentration of methylmercury (MeHg) is involved in GSK-3β overexpression [58]. Environmental neurotoxicant Arsenic (As) has been reported to increase GSK-3β kinase activity [54]. MPP+ (1-methyl-4-phenylpyridinium) treated cultured cells (PC12) of rat adrenal medulla displayed the increased Dkk1 expression and inhibition of the canonical Wnt pathway [59]. An emerging neurotoxin, antimony (Sb), upon treatment on rat PC12 cells, causes β-catenin suppression and GSK-3β activation [53]. Furthermore, administration of silica nanoparticles (SiNPs) in human SK-N-SH cells has shown a significant reduction in phosphorylation of GSK-3β that increases its kinase activity resulting in pathological Aβ deposition and increased tau phosphorylation [62].

##### Dysregulation of autophagy and mTOR signalling by neurotoxicants

Autophagy is an intracellular multiplex sequence mediated by vesicles and lysosomes that efficiently digest and recycle damaged and misfolded proteins and dysfunctional organelles, thereby sustaining cellular homeostasis. Neuronal endurance and activities are intensively reliant on the productive expulsion of damaged proteins and defective organelles. Unlike non-neuronal cells that require a low basal level of autophagy, matured neuronal cells are post-mitotic, i.e. they are unable to undergo mitosis [84] in response to damage by toxic substances leading to their dependency on the high basal rate of autophagy [85]. Although autophagy plays a defensive role in the nervous system by clearing destructive protein aggregates and malfunctioned organelles, exacerbated autophagy damages tissues and cells, and hyperactivation of autophagy results in neuronal death [63]. Among the three types of cell death, type 2, or autophagic cell death, is marked by the accumulation of multiple autophagosomes and autolysosomes, where organelles are destroyed in autolysosomes. It is reported that the nucleus and cytosolic filaments remain unharmed; however, the nucleus eventually gets demolished by lysosomal hydrolases in some instances [86]. Regarding the pathological process of many neurodegenerative diseases, autophagy is perceived as a two-edged sword [87]. Several autophagic deregulations, including interference with the association of autophagosome and lysosome, depletion of lysosomal acidification or presence of cellular protein stockpile, etc., are involved in the pathological mechanism of neurodegeneration [85,87]. Brain autopsy of AD patients revealed the overabundance of autophagosomal and pre-lysosomal vacuoles in neuronal dendrites, along with indications form of both AD animal models and AD patients strongly support the fact that dysregulation of autophagy or autophagosomal disposal is a potential mediator of Alzheimer’s disease [88,89].

**Figure 4.**
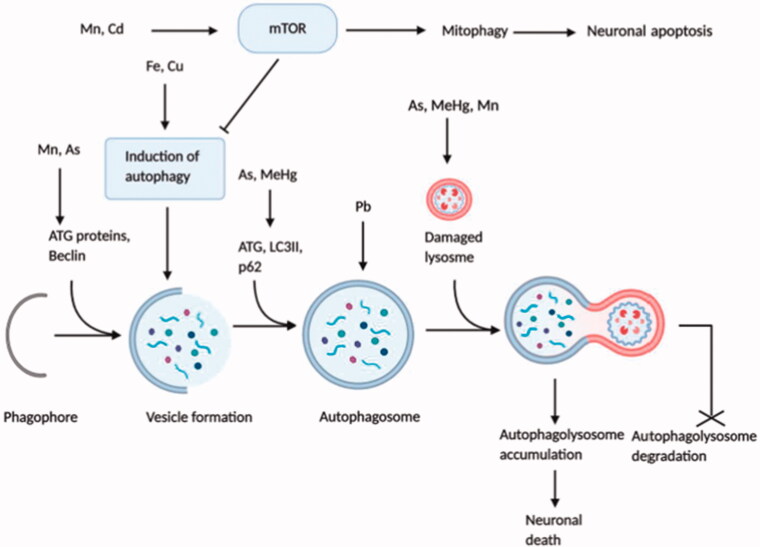
Dysregulation of autophagy and mTOR signalling by neurotoxicants. Although autophagy and mTOR signalling are vital for the healthy and normal functioning of neurons, some neurotoxicants interrupt their regulation. Excessive activation of mTOR signalling through neurotoxicants (Mn, Cd) results in mitophagy and neuronal apoptosis that inhibits normal autophagic function. However, autophagy-related proteins, including ATG, Beclin, LC3II, etc. and different autophagic steps are negatively influenced by neurotoxicants (Fe, Cu, Mn, As, Pb, MeHg), leading to the uncontrolled autophagic influx. Some neurotoxicants (As, MeHg, Mn) damage lysosomal structure, which after fusion with autophagosome, produces immature autophagolysosome vacuoles (AVs) and halt the degradation of autophagolysosomes. Finally, increased accumulation of AVs in neurons triggers neuronal death.

Mainly autophagy is regulated according to the physiological condition of cells, and the protein kinase mTOR acts as a principal inhibitor in autophagy regulation through different pathways [86]. Some signalling molecules such as 5′-adenosine monophosphate-activated protein kinase (AMPK), PI3k-Akt (phosphatidylinositol-3-kinase and protein kinase B) are interlinked with mTOR function and autophagy regulation [63,86]. In neurons and glial cells, mTOR proteins are highly expressed, and their modulatory activities are fundamental in brain development. In the adult brain, mTOR signalling plays a crucial role in the translational initiation of protein synthesis required for synaptic plasticity and memory formation. However, exaggerated protein synthesis interposed by uncontrolled mTOR activity leads to impaired plasticity and memory deficits and neurological disorders. Extensive analysis of autopsied AD brain and AD mice model revealed the connection between deregulated mTOR signalling and neurodegenerative diseases. Various autophagy-related proteins arbitrate the three different steps of autophagy, i.e. initiation, elongation, and maturation; in which the formation of a membrane structure in the cytoplasm called phagophore initiate the first step while the maturation step is mediated by the fusion of the autophagosome with an endosome or lysosome, and finally, lysosomal enzymes degrade the autophagosome cargo. In normal cellular conditions, activated mTOR kinases inhibit autophagy-related proteins; furthermore, they disrupt autophagosome formation. Some evidence specified that mTOR proteins negatively regulate autophagosome formation and lysosomal biogenesis through a transcription factor EB (TFEB) phosphorylation associated with the lysosomal function [90]. Overexposure to an assortment of toxic metals and essential metals triggers cellular stress responses that deregulate the autophagic pathway contributing to neurological damage (Figure 4) [87].

Accumulation of essential metal, Fe, in cultured neuroblastoma cells induced the oxidative stress response that stimulated autophagy activation [66], while another experiment showed that autophagy-mediated by oxidative stress triggered Fe accretion in the cell [91], making Fe accumulation as both the source and result of autophagy following oxidative stress [92,93]. It has appeared that Cu triggers autophagy both in cancer cells and neurons [87]. In the catecholaminergic cell line, conjugated Cu and dopamine gave rise to mitophagy leading to apoptotic cell death [94]. Practical exposure to Cu enhanced autophagic influx and deteriorated autophagic activities that led to Cu-induced dopaminergic cell death [65]. Cadmium (Cd), an industrial pollutant, disrupts several intracellular mechanisms, and Cd-mediated cytotoxicity is regarded as a potential causative factor of neurodegenerative diseases such as AD [87]. Cd elevates intracellular Ca^2+^, followed by mTOR signalling activation that ultimately results in neuronal death by apoptosis [64,95]. Several studies indicated apoptosis as an immediate outcome of autophagic stimulation [87]. Additionally, Cd enhances the over-activation of mitophagy that causes cellular energy depletion followed by cell death [96,97]. Pb, a predominant environmental neurotoxicant, has proved to be involved with autophagy induction in both *in vitro* and *in vivo* systems [87]. Research from a rat model displayed that long-term exposure to drinking water containing Pb upregulated autophagosome formation and excessive autophagy induction contributed to cell death [70]. The autophagic process was found dysregulated in a rat model after Mn exposure causing neuronal death. During early exposure (4-12 hr), autophagy was elicited, as evidenced by increased expression of autophagy-related proteins, i.e. Beclin1 and LC3II; where a long term exposure (1–28 days) led to inhibited autophagy, displayed by an enhanced level of unusual lysosomes, reduced level of Beclin1 and LC3II along with activated mTOR signalling [68]. Another *in vitro* study on astrocytic glial cells demonstrated that in response to short-term Mn exposure autophagy was activated for cytoprotection, although long-term exposure promoted lysosomal membrane permeabilization and defected autophagy with subsequent glial cell death [69]. Experimental mouse cerebellum showed that As exposure suppressed the mTOR protein levels while induced the expression of autophagy biomarkers (Beclin1, LC3 and Atg12) that initiated autophagy. But an increase in p62 level was found that is a marker of dysfunctional autophagic influx [63]. MeHg has been found to be a causative factor of neuronal cell death. LC3II was enhanced due to MeHg exposure which activated autophagy; meanwhile, MeHg also upregulated p62 expression. MeHg affected lysosomal permeability as well. All of which resulted in MeHg induced cell death [67].

Despite having several observations on the effect of neurotoxicants in defected autophagy and mTOR signalling, the exact mechanism remained elusive since there is no linearity among all these incidences.

##### Alteration in PKC signalling by neurotoxicants

The protein kinase C, part of an integral brain signalling network, comprises serine/threonine protein kinases of about 12 mammalian isoforms, also known as the “memory kinases” [98]. They are divided into three sub-division based on their structure and fastidious response to second messengers: i) Conventional or calcium-dependent PKC (cPKC: α, βI, βII, and γ) – required Ca^2+^ and DAG for activation; ii) Novel PKC (nPKC: δ, ε, η, μ, and θ) – calcium-independent and needed DAG; iii) Atypical PKC (aPKC: λ, ζ, ι, and PKMζ)- no regulatory domain [98,99]. Multiple neuronal functions, including cellular growth, differentiation, metamorphosis, synaptic function, and behavioural conscience, are regulated by neuronal signal transduction, where reversible substrate protein phosphorylation catalyzed by PKC is an essential mechanism [98].

**Figure 5.**
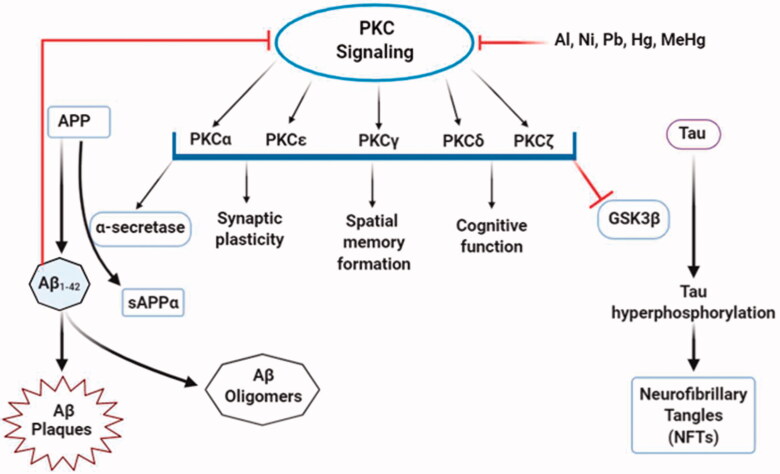
Alterations in PKC signalling by neurotoxicants. PKC signalling is associated with multiple functions, including the formation of sAPPα and inhibition of GSK-3β that decreases Aβ production and tau hyperphosphorylation. At the same time, some neurotoxic agents (Al, Pb, Ni, Hg, MeHg) interfere with PKC enzyme expressions and activities that alter normal PKC signalling. This, in turn, enhances Aβ_1–42_ production and directly affects PKC signalling.

In the brain, the protective role of PKC depends on different PKC isoforms since different isozymes have distinct as well as opposite modulatory functions [99]. PKCα, PKCε, PKCγ, PKCδ, and PKCζ, in particular. are strongly associated with cognitive functions and disorders. In the hippocampus, activation of PKCα and PKCε stimulates the expression and release of neurotrophic factors, followed by high impact and spatial memory formation and learning abilities [98]. Contrastingly, over-activated PKCα and PKCδ isoforms can lead to loss of dendritic function and psychological dysfunctions. PKCε and PKCγ are also involved in the progression and maintenance of synaptic formation and plasticity. Enhanced activation of PKCζ is crucial for long-term memory development and storage [100]. Besides their memory and cognitive function involvement, PKC isoforms control some pathophysiological pathways related to AD [101]. The activation of PKCα and PKCε mediates cleavage of APP by α-secretase while Aβ inhibits PKC isozymes [102]. The α-secretase product sAβPPα fosters PKCβ translocation assisted by RACK1 into the plasma membrane, but disruption in PKCβ translocation causes tau hyperphosphorylation resulting in AD pathology. On the other hand, activated PKCε attenuates Aβ aggregation by the degradation and clearance of Aβ [100]. Among various kinases that phosphorylate tau, GSK-3β is the most prominent, and PKC regulates tau phosphorylation and NFT formation by direct inhibition of GSK-3β [99,102]. The mechanistic role of PKC in AD aetiology was observed from many pieces of evidence that found a systematic deficiency and alteration in PKC signalling in postmortal AD brains and transgenic animal models [103]. Besides, growing evidence demonstrated the adverse effects of some neurotoxicants in the PKC signalling pathway (Figure 5).

Toxic heavy metals, including Al, Ni, and Pb, have inhibitory effects on PKC signalling [73]. *In vivo* and *in vitro* experiments on rodent models showed a significant decrease in PKC concentrations after aluminium lactate consumption [71]. But, another animal model suggested an increased PKC activity by aluminium sulphate oral doses [104]. These contrasting effects may be due to variation in aluminium concentration following its consumption and delivery to the brain and exposure length. PKC activities through gene expression studies from PC12 cells detected transcription of two PKC isoforms (PKCγ and PKCζ), and two adapter proteins, RACK-1 (receptor for activated C kinase 1) and PKC-δ binding protein were reduced upon Ni^2+^ exposure [75]. An *in vitro* study has indicated that at low concentrations, heavy metals, including Hg, Pb, and MeHg retard the function of PKC enzymes, and MeHg is the most potent inhibitor [74]. At higher concentrations, Pb interferes with the catalytic domain of PKC enzymes and inhibits PKC activation [75]. It has been shown to stimulate PKC through Ca^2+^ substitution. Despite that, a neuron culture study revealed that Pb^2+^ had a stimulatory effect on PKC at low concentrations and a repressive effect at high concentrations, making Pb a partial agonist of PKC [72].

However, it is not the function of PKC deficient in AD; somewhat, the expression level, the kinase activity, and their translocation to subcellular organelles are also affected [99]. It can be deduced from the above discussion that neurotoxicants are involved in each of these occurrences. Therefore, more comprehensive research on this subject is essentially required.

#### Neurotoxicants and cellular stresses

Cellular oxidative stress is induced when there is a discrepancy between the exemplification of free radicals, reactive oxygen species, and the detoxification process of biological antioxidants, which is implicated in neurological diseases. The human brain is deemed as the gateway of reactive oxygen/nitrogen species (ROS/RNS) and free radicals because it uses a substantial amount of oxygen for functional efficiency [105]. Even though free radicals, ROS, and RNS play a crucial function in multiple cell signalling pathways (cell cycle regulation, phagocytosis, and enzyme activations), they have damaging effects on the normal function and oxidative state of essential biomolecules, including nucleic acids, proteins, and lipids leading to mitochondrial deficits, DNA/RNA damage, misfolded proteins, lipid peroxidation, dysfunctional energy metabolism as well as neuronal death [105,106]. Simultaneously, heavy inorganic metals, organic chemicals, and other xenobiotics are known as potent inducers of oxidative stress by altering the cell’s redox status and negatively influencing the biomolecules of the vital organs, including the CNS (Table 2). The harmful effects of oxidative imbalance such as oxidative damage of DNA, RNA, and proteins, lipid peroxidation, reduced antioxidative enzyme activity, metal dyshomeostasis, and mitochondrial aberration are responsible for neuronal injury are predominant features in AD [105]. For the absorption of essential metal ions into cells, several cellular transporters and carrier proteins are recruited. On the other hand, toxic metal ions exploit these transporters and carrier proteins to gain access inside cells through molecular mimicry. Furthermore, due to the lipid bilayer membrane’s composition, lipophilic non-polar organic substances can easily permeate into the cytoplasm [127]. During normal physiological conditions, electron transport chains (ETC) of mitochondria use up 98% of oxygen, and the remnants are converted to free radicals while exaggerated free radical generation induces cellular stress. The toxic ROS, in turn, causes impaired mitochondrial function. In the context of neurodegenerative diseases, especially in AD, mitochondria is both the origin and victim of oxidative stress [128].

**Table 2.** Role of neurotoxin-induced cellular stresses in the progression of Alzheimer’s disease.

Sl	Neurotoxic agent	Study type	Molecular targets	Key modulating effects	References
Toxic heavy metals					
1	Aluminium (Al)	*In vitro* *In vivo*	Hippocampal and frontal brain	Interruption in biometal homeostasis in neuron cells that produce oxidative damage; alteration in signalling pathways that promote neuropathology	[107]
		*In vitro* *In vivo*	Neurons	Induction of oxidative stress that stimulates Aβ and NFT accumulation	[108,109]
		*In vitro* *In vivo*	Aβ catalysing enzyme and antioxidant PP2A enzyme	Inhibition of Aβ catabolizing enzyme and PP2A enzyme activities; reduction of Aβ degradation; stimulation of NFT formation	[110,111,112]
2	Arsenic (As)	Literature review	Glutathione (GSH)	Binding with –SH groups of glutathione; production of excessive H_2_O_2_	[107]
4	Cadmium (Cd)	*In vitro*	Ca^2+^	Disruption of cytoplasmic and nuclear Ca^2+^ homeostasis; modulation of cellular processes	[113]
		*In vitro*	Antioxidant enzymes: GSH, catalase, and SOD	Inhibition of GSH, catalase, and SOD activities; induction of cellular oxidative stress and lipid peroxidation	[114]
4	Calcium (Ca)	Literature review	Enzymatic process	Lipid peroxidation, protein destruction and neuronal death promoted by excessive intracellular Ca influx	[115]
5	Copper (Cu)	*In vitro*	Aβ	Formation of Cu-Aβ conjugate; production of H_2_O_2_	[107]
		*In vivo*	τ-protein	Induction of H_2_O_2;_ formation of Aβ and hyperphosphorylated τ	[116]
6	Iron (Fe)	Literature review	CNS	Overproduction of ROS due to high Fe concentration;	[107]
		Literature review	Mitochondria	Induction of oxidative stress; disruption of mitochondrial function; initiation of cytotoxic reactions.	[117]
7	Lead (Pb)	*In vitro*	DNA methylating enzymes	Decreased expression of DNA methylating enzymes that enhance APP expression and attenuates Aβ elimination	[107]
		Literature review	Antioxidant enzymes and metal cofactors	Binding to metal cofactors and –SH groups of antioxidant enzymes; disruption of various processes which elevate oxidative stress and mitochondria dysfunction	[105]
11	Manganese (Mn)	*In vitro* *In vivo*	Ca^2+^ and mitochondria	Obstruction of Ca^2+^ efflux in mitochondria; impediment in mitochondrial Ca^2+^ homeostasis	[117]
		Literature review	CNS	Increased ROS production; impaired mitochondrial function; reduction in cellular antioxidant mechanism	[107]
		*In vitro*	Mitochondria	Inhibition of ETC; reduction in oxidative phosphorylation and ATP formation; disruption in mitochondrial membrane permeability	[117]
8	Mercury (Hg)	*In vitro*	Neuron cells	Induction of oxidative stress *via* ROS production; increased Aβ and τ pathology	[118]
10	Methylmercury (MeHg)	*In vitro* & Literature review	–SH group containing proteins and antioxidative enzymes	Alteration in the oxidative state of –SH containing proteins and antioxidant enzymes; modulation of their function	[117]
11	Zinc (Zn)	Literature review	ETC	Intervention in ETC complex-III; stimulation of ROS production; induction of mitochondrial dysfunction and neuronal death	[119]
Pesticides and herbicides					
1	Organochlorines, organophosphates and bipyridal herbicides	*In vivo* & literature study	CNS	Generation of free radicals that increases cellular oxidative stress	[120,121]
Industrial chemical					
1	Bisphenol-A (BPA)	*In vitro*	Neurons	Disruption in intracellular Ca^2+^ homeostasis, endoplasmic reticulum and mitochondrial dysfunction; decreased level of antioxidant enzymes, e.g. GSS and CAT	[122]
		*In vivo*	CNS	Damaged neurons and organelles	[123]
			Functional proteins	Lipid peroxidation and alteration in proteins expression level	[124]
Nanoparticles					
1	Copper nanoparticles (nano-CuO)	*In vitro* *In vivo*	Antioxidant enzymes – SOD and GSH-Px	Production of ROS and MDA; inhibit SOD and GSH-Px activity	[125]
2	Silica nanoparticles (SiNPs)	*In vitro*	Neuronal cell	Production of intracellular ROS; induction of apoptosis	[62]
3	Titanium dioxide nanoparticles (TiO_2_ NPs)	*In vitro* *In vivo*	Hippocampal cell	Induction of cellular oxidative stress, glial reactivity and apoptosis	[126]

In the ageing brain, cellular damage is most likely to be caused by extreme Fe levels linked with ROS production [107]. Multiple experimental data illustrates that Fe is responsible for the aetiological process of neurodegenerative since it produces oxidative stress, disrupts mitochondrial function, and initiates cytotoxic reactions [117]. Several enzymatic processes, i.e. lipid peroxidation, protein destruction, and neuronal death, are resulted from excessive intracellular Ca^2+^ influx [115]. At the same time, Cd disrupts cytoplasmic and nuclear Ca^2+^ homeostasis in neurons that alters some cellular processes [113]. In the brain, Cd induces ROS and inhibits some antioxidant enzyme activities such as glutathione (GSH), catalase, and superoxide dismutase (SOD) thereby, contributing to cellular oxidative stress [114]. Al builds up in the hippocampal and frontal brain region, where it impedes biometal homeostasis resulting in oxidative stress and alters signalling pathways, thereby promoting neuronal death and neuropathology. Neurotoxic effects of Al have been observed in both *in vivo* and *in vitro* models, and the outcome demonstrates that increased oxidative stress-mediated by Al promotes Aβ and neurofibrillary tangle (NFT) accumulation [107]. Al interferes with Aβ catabolizing enzymes facilitating the amyloidogenic pathway [110,111], and decreases Aβ degradation; also inhibits protein phosphatase 2 (PP2A) activity that triggers NFT deposition [112]. Exorbitant Zn exposure causes impediment of electron transport chain (ETC) complex-III and stimulates ROS production, which in turn leads to mitochondrial dysfunction and neuronal death [119]. In an animal model, Cu induces H_2_O_2_ formation that accelerates both formation of Aβ and the hyperphosphorylation of *τ* [116].

Neurotoxicity of Hg coincides with increased oxidative stress by ROS production that triggers Aβ and τ pathology [118]. MeHg binds with –SH group-containing proteins and antioxidative enzymes, alters their oxidative state, and, finally, weakens their activities [117]. Pb exposure on cultured cells has demonstrated that the downregulation of DNA methylating enzymes attenuates Aβ elimination [107]. Due to its high affinity for –SH groups and metal cofactors, Pb binds with different antioxidant enzymes and disrupts various biological processes, elevating oxidative stress and mitochondrial dysfunction [105]. Similarly, the toxic metalloid, As, also binds to the –SH group of glutathione (GSH), resulting in excessive H_2_O_2_ production [107]. Neurotoxicity of Mn is associated with ROS production, impaired mitochondrial function, and reduced cellular antioxidative mechanisms [107]. Mn obstructs Ca^2+^ efflux in mitochondria and interrupts mitochondrial Ca^2+^ homeostasis. Oxidative stress generated by elevated Mn levels inhibits ETC, reduces oxidative phosphorylation and ATP production, disrupts mitochondrial membrane permeability [117]. BPA disrupts intracellular Ca^2+^ homeostasis leading to neuronal ER and mitochondrial malfunction. BPA-induced oxidative stress is caused by exacerbated ROS generation and decreased levels of antioxidative enzymes such as glutathione synthetase (GSS) and catalase (CAT) [122]. Neurons, as well as cellular organelles in the brain hippocampus, can be damaged by BPA-induced oxidative stress [123]. Besides, BPA triggers lipid peroxidation and alteration in protein profiles whose function is crucial for neurons’ metabolic processes. These proteins’ decreased level and activity are interlinked with neurodegenerative and psychotic disorders [124]. A wide range of pesticides and herbicides such as organochlorines, organophosphates, and bipyridyl herbicides have long been known to generate free radicals and increase cellular oxidative stress as a mechanism of their neurotoxic effects [120,121].

Speculative indications are rising on the prospective role of nanoparticles in neurological disorders. In a rat model, administration of copper nanoparticles (nano-CuO) generates ROS and malonaldehyde (MDA) while undermining the functionality of antioxidant enzymes, superoxide dismutase (SOD) and glutathione peroxidase (GSH-Px) in the hippocampus that hampers cognitive function in the experimented animals [125]. Another animal model has shown that titanium dioxide nanoparticles (TiO2NPs) increase cellular oxidative stress, glial reactivity, and apoptosis in hippocampal cells, which is associated with traumatic cognition [126]. Human SK–N–SH and mu rine neuro2a (N2a) neuroblastoma cells were treated to SiNPs in a cell culture paradigm. The results of this experiment show that SiNP is a viable option.

#### Neurotoxicants and glutamate receptors (iGluRs and mGluRs)

The most common excitatory neurotransmitter, glutamate, and its receptor, which are required for neuronal cell differentiation, migration, survival, and synaptic plasticity, conduct a large amount of excitatory neurotransmission in the human CNS. There are two types of glutamate receptors:-ionotropic and metabotropic receptors. Three ionotropic glutamate receptors have been identified: NMDA, AMPA, and Kainate receptors (KARs), as well as three metabotropic glutamate receptors. iGluRs and mGluRs are two types of glutamate receptors. Over-activation of these receptors by some neurotoxic substances, on the other hand, causes neuronal excitotoxicity as well as neuronal death, which could be a key mechanism of neurodegeneration in Alzheimer's disease (Table 3) [145]. Kainic acid (KA) is an agonist for the iGluR subtype, and it causes an increase in reactive oxygen species generation, mitochondrial malfunction, and death in neurons across the brain [136]. DomA (3,4-dihydroxymandelic acid) is a structural counterpart of KA, a neurotoxic produced naturally by diatoms in the sea. Glutamate binds to both iGluRs and mGluRs and activates them. In the brain, intracellular glutamate concentrations remain millimolar, while extracellular glutamate concentrations remain micromolar. These quantities are maintained by excitatory amino acid transporters, which import glutamate and aspartate into astrocytes and neurons. Excess extracellular glutamate promotes excitotoxicity by over-activating iGluRs [146]. Glutamate excitotoxicity is associated with cell death, apoptosis, and autophagy in hippocampus cells (HT22) and primary cultured hippocampal neuron cells, as well as neurotoxicity in differentiated Y-79, BV-2, and PC12 cells. Mitochondrial malfunction, oxidative damage, and neuroinflammation are some of the harmful outcomes of glutamate-induced neurotoxicity [25,130–132]. Homocysteine is a neurotoxic agent in Alzheimer's disease, causing synaptic malfunction, oxidative stress, neurochemical imbalance, and apoptosis, all of which lead to cognitive impairment and neuronal cell death [133,134]. The non-proteinogenic amino acid β-N-methylamino-L-alanine (BMAA) is a common neurotoxin which causes neurotoxicity by acting as an agonist for glutamate receptors such as AMPARs/KARs, NMDARs, and mGluR5 [129]. Another neurotoxicant, called Harmaline, modulates both iGluRs and mGluRs. Harmaline is a mGluR1 agonist that causes motor abnormalities [137]. Ammonia is also a neurotoxicant that modulates glutamate receptors and activates NMDARs, resulting in the following effects: (i) depletion of adenosine triphosphate (ATP) in the brain causes glutamate release; (ii) activation of calcineurin, dephosphorylation, and Na+/K+-ATPase in the brain increase ATP consumption; (iii) impairment of mitochondrial function and calcium homeostasis at different levels reducing ATP synthesis; (iv) activation of calpain degrades microtubule-associated protein-2 alters the microtubular network; and (v) rising the formation of NO reduces the activity of glutamine synthetase, thus decreases the elimination of ammonia in the brain [138]. In an experimental model, synaptic transmission was affected by H_2_O_2_, where oxidative stress was promoted through the activation of NMDARs [141]. By raising NMDAR expression and responsiveness, Hg causes various negative effects such as mitochondrial dysfunction, oxidative stress, neuroinflammation, and apoptosis, culminating in cognitive impairments and neuronal cell death [28,142,143]. Sodium Azide is a neurotoxic substance that activates NMDARs and kills neurons [144]. BPA causes neurotoxicity through altering glutamate receptors such as NMDARs and AMPARs [138,139,147, , ].

**Table 3.** Effects of neurotoxic agents on iGluRs and mGluRs in the onset and progression of Alzheimer’s disease.

SL	Neurotoxic agents	Study type	Molecular targets	Key modulating effects	References
Excitatory amino acids					
1	BMAA	*In vitro* *In vivo*	AMPAR/KAR, NMDAR, mGluR5	Neuroinflammation, oxidative stress, apoptosis, cognitive impairment	[129]
2	Glutamate	*In vitro* *In vivo*	iGluR and mGluR	Induction of apoptosis, autophagy mitochondrial dysfunction, oxidative damage and neuroinflammation.	[130,131,132,25]
3	Homocysteine	*In vivo*	NMDAR, mGluR1	Synaptic dysfunction, oxidative stress, neurochemical imbalance, apoptosis/necrosis, neuronal cell death	[133,134]
Excitatory amino acid agonist					
1	Domoic acid (DomA)	*In vivo*	iGLuR	Neuroinflammation, mitochondrial dysfunction, production of ROS	[135]
2	Kainic acid	*In vitro* *In vivo*	iGluR	Production of ROS, mitochondrial dysfunction, neuroinflammation and neuronal autophagy	[136]
CNS stimulant					
1	Harmaline	*In-vivo*	NMDARs, AMPARs and mGluR1	Disturbance in motor function, production of tremors	[137]
Industrial organic compound					
1	Bisphenol-A	*In vitro*	NMDAR, AMPAR	Production of neurotoxicity	[138,139,139]
Inorganic compound					
1	Ammonia	*In vivo*	NMDAR	Reduction of glutamine synthetase activity, decreased elimination of ammonia from the brain	[140]
2	Hydrogen peroxide	*In vitro*	NMDAR	Alteration in synaptic transmission, oxidative stress, mitochondrial dysfunction, cytotoxicity, apoptosis, neuronal cell death	[141]
3	Mercury	*In vitro*	NMDAR	Mitochondrial dysfunction, oxidative stress, neuroinflammation, apoptosis, neuronal cell death	[28,142,143]
4	Sodium azide	*In vitro*	NMDAR	Mitochondrial dysfunction, oxidative stress, neuroinflammation, neuronal cell death	[144]

#### Neurotoxicants and immunoirregulations

Neuron cells are the brain’s central functional unit, mediating electrical and chemical signal transduction and transmitting information to different parts of the body by connecting with one another. Glial cells, which come in a variety of shapes and sizes, such as microglia, astrocytes, and oligodendrocytes, assist the brain stay healthy by supporting and shielding neurons from damage. Microglia is the most important immune cell in the central nervous system, and it functions as a stimulant for the immune and neuronal cells that surround it [148]. Due to extreme sensitivity, microglia become activated in response to multiple stimuli such as pathogens, toxic substances, air pollutants, abnormal protein aggregates, or even minor brain damage and play a prime role in initiating neuroinflammation [149–151]. Following these triggers, microglia generates a wide range of cytotoxic factors, including interleukins, TNF-α, superoxide, nitric oxide, ROS, and prostaglandins, that eventually lead to neuronal death [152]. Increased microglial activity and its toxic effects have long been incriminated in the pathological process of Alzheimer’s disease [150].

Toxic metals, pesticides, nanoparticles, and antimicrobials are associated with the aggregation and stabilization of abnormal Aβ protein, hyperphosphorylated tau (τ) protein, and disturbance in cellular redox potential [18,149,153,154]. Furthermore, these neurotoxicants directly damage various neuronal cells because of their chemical nature; they also indirectly harm the brain through increased glial reactivity [151]. Accumulation of neurotoxicants [17] and imbalance of essential metals in extracellular areas [155] interfere with immune cell surface receptors and activate microglia, astrocytes, macrophages, and lymphocytes that produce pro-inflammatory cytokines, including 1 L-1, IL-8, TNF-α, IL-6 and ROS [156].

Air pollutants, a multidimensional causative factor, have been linked with neuroinflammation and AD. They are a mixture of granulated molecules, such as CO, SO2, NO, O3, heterocyclic aromatic hydrocarbons, Ni, Mn, vanadium, endotoxins, etc. Investigations from human and animal brains revealed that air pollutants activated microglia and monocytes, promoted pro-inflammatory cytokine (IL-1β and COX2) production, and enhanced Aβ deposition [157]. *In vivo* and *in vitro* studies on animal models indicate that TMT (2,4,6-Trimercapto-1,3,5-triazine) is responsible for microglial reactivity, producing pro-inflammatory molecules, i.e. IL-1β, IL-12, IL-23, TNF-α, and NO and neuronal death, especially in the hippocampal region [152]. Another *in vitro* study on neurons has shown that Hg triggers glial cell activities that cause neuroinflammation [158]. Some pesticides include rotenone, lindane, dieldrin, PQ, etc. possess stimulatory effects on microglial activation and neurotoxic substance productions that result in chronic neuroinflammation [150]. A joint inflammatory agent, LPS (lipopolysaccharide), has been shown to induce microglial activation in both *in vivo* and *in vitro* models. After activation, microglia cells yield neurotoxic molecules and ROS [159].

Microglial activation paves the way for astrocyte reactivity, where secreted cytokines play a significant role in cell-cell signalling between them, causing inflammation [151]. Microgliosis and astrogliosis are prominent features of Alzheimer’s disease. Strongly controlled regulation of astrocytes and microglial activity regarding immune challenges preserves brain homeostasis and healthy neuronal function. At the same time, chronic stimulation causes excessive pro-inflammatory reactions resulting in the over-activation and deregulation of these immune cells, which contribute to pathogenic neuroinflammation and neurodegeneration attributed to Alzheimer’s disease [160]. Sustained immune response by activated microglia and other immune cells has been revealed to promote amyloid and tau pathology by releasing proinflammatory cytokines [161]. Chronic neurotoxicant-mediated injury and Aβ aggregation diminish the activity of microglia to clear the toxic protein components though its pro-inflammatory cytokine production remains unchanged [162,163], which in turn activates more microglia and peripheral macrophages to the site of Aβ aggregates [161]. As a result, the continuous activation of microglia and the secretion of pro-inflammatory cytokines and microglial neurotoxins, which is known as reactive microgliosis, leads to an exaggerated inflammatory response that aids neurodegeneration [162]. Among the inflammatory cytokines, IL-1 improves the development of Aβ through amyloidogenic proteolysis of APP and increases the production of other cytokines such as IL-6 and TNF-α [161]. TNF-α and other cytokines are produced by the NF-κB dependent pathway, where the transcription factor NF-κB is activated by the interaction of neurotoxicants with microglia and astrocytes [164,165] and accumulation of abnormal proteins in neuronal cells [161]; meanwhile, TNF-α sets off Aβ production by upregulating the production of APP cleaving enzyme β- secretase and increment of γ-secretase activity [166,167] and IL-6 activates a kinase, CDK5 that hyperphosphorylate tau protein [168]. Ultimately, a vicious cycle is established in which neurotoxicant-induced oxidative stress and atypical protein accumulation stimulate principal immune cells, i.e. microglia and astrocytes causing the development of proinflammatory cytokines, which intensifies the Aβ and tau pathology associated with AD.

#### Neurotoxicants and epigenetic modulation

Mutations in APP, PSEN1, and PSEN2 genes cause the early onset of Alzheimer’s disease [169]. An explicit form of Barker’s hypothesis, Developmental Origin of Health and Disease (DOHaD), stated that maternal diet, malnutrition, smoking, and stress during prenatal stages might be linked to adult life diseases, including cancer, metabolic disorders, neuroendocrine and neurodegenerative diseases. A mechanical hypothesis showed that the number of neurons in crucial brain areas is decreased due to early life exposure to neurotoxicants, initiating a self-sustaining neurodegeneration process [154]. Sporadic cases of AD indicating environmental factors can navigate the formation of AD pathology has been a subject of long dispute because the mechanism in which it occurs is not yet known; however, some *in vivo* and *in vitro* studies have suggested that environmental factors such as toxic metals, air pollutants, etc. in developmental stages change the expression and regulation of late-life APP [170].

In the brain, an excessive amount of Al alters the function of APP, PSEN1, and PSEN2 genes [107]. An experiment showed that chronic oral administration Al enhances APP expression in rat hippocampus and cortical tissues [171], increasing the insoluble aggregation of Aβ protein. Furthermore, a research group suggested that toxic metals disrupt micro RNA (miRNA) homeostasis, potentially biomarkers of AD pathogenesis [172]. Al exposure in mice increases the expression of miRNAs linked to pro-inflammatory responses, which were similar in AD [173,174]. Human astroglial cells treated with Al and Fe resulted in the induction of NF-κB mediated miRNAs expression reported in AD pathology [175,176]. Extracellular Aβ deposits contain a high amount of Fe that facilitates APP expression as well as Aβ formation. Iron responsive elements (IREs) are found within the 5′-UTR region of APP mRNA, while increased Fe levels bind to this region and enhance APP gene transcription and translation [107]. Mn exposure chronically alters amyloid-β precursor-like protein1 (APLP1) gene expression that remains highly upregulated in the frontal AD brain of non-human primates [177]. Chronic exposure to a high amount of Ca in the brain augments APP and APOE gene expression and Aβ aggregation by stabilizing the γ-secretase enzyme [178]. In rodents, developmental exposure of Pb from birth to post-natal days led to APP upregulation and a subsequent rise in Aβ plaques in later life [179]. Overexpression of AD-related genes including APP, β-site APP cleaving enzyme1 (BAEC1), and their transcriptional regulator (Sp1) and the onset of AD pathogenecity was observed in aged primates 96% sequence homology with humans. They were exposed to Pb in childhood which caused a decrease in DNA methyltransferase activity and increases in DNA oxidative damage in adolescence, showing epigenetic alteration in AD-related gene expression [170]. *In vitro* studies also showed that exposure elevates APP expression and insoluble Aβ protein levels [149]. Rats subjected to a mixture of As, Cd, and Pb from gestational day-5 to postnatal day-180 exhibited a dose-dependent increase in Aβ protein through enhanced amyloidogenesis, which is mediated by upregulated expression of APP, and subsequent β-secretase enzyme (BACE), and presenilin (PS)-mediated APP processing in the frontal cortex and hippocampus area [180]. A cultured cell experiment has revealed that Hg upregulates APP expression and insoluble Aβ formation [158]. Experiments on human and rat cultured cells upon SiNPs led to the enhanced expression of APP genes while reduced the expression of the Aβ degrading enzyme, neprilysin [62]. Similarly, silver nanoparticles (AgNPs) increase APP gene expression and downregulate neprilysin (NEP) and low-density lipoprotein receptor (LDLR) expression that contributes to Aβ plaque aggregation [181].

## Concluding remarks

Alzheimer’s disease appears to have a complex mechanism, as evidenced by its innumerable clinical manifestations. Researchers are currently attempting to determine which factor plays the most critical role in AD origination. Many pathogenic factors, including extracellular Aβ aggregates, intracellular tau (τ) entanglements, synaptic and cholinergic malfunctions, inflammation, and substantial oxidative stress, are prominent AD features. A plethora of evidence supports the notion that many neurotoxicants are directly connected to the pathological progression of AD. Specifically, these neurotoxicants are attributed to the dysregulation of numerous signal transduction pathways, cellular stress induction, neuroinflammation, immunoirregulations, genetic misappropriations as well as neuronal death. Eventually, the findings from the review revealed the longstanding correlation between neurotoxicants and AD pathophysiology, which can be taken into consideration in future extensive researches for the management of AD and related neurodegenerative disorders.

## Data Availability

Data available on request from the authors.

## List of abbreviations

4-HNE: 4-hydroxy-2-nonenal
a7nAChR: nicotinic acetylcholine receptors
AchE: acetylcholinesterase
AD: Alzheimer's disease
ADDL: Aβ-derived diffusion ligands
AgNPs: silver nanoparticles
Al: aluminium
AMPA: α-amino-3-hydroxy-5-methyl-4-isoxazolepropionic acid
AMPARs: α-amino-3-hydroxy-5-methyl-4-isoxazolepropionic acid receptor
AMPK: 5’-adenosine monophosphate-activated protein kinase
antiACHs: anticholinesterases
APLP1: amyloid-β precursor-like protein-1
APOE4: apolipoprotein E4
APP: amyloid precursor protein
As: arsenic
Aβ: amyloid-beta
BAEC1: β-site APP cleaving enzyme1
BBB: blood brain barrier
BDNF: brain derived neurotrophic factor
BMAA: β-N-methylamino-L-alanine
BPA: bisphenol-A
Ca: calcium
CAT: catalase
Cd: cadmium
CD36: cluster of differentiation 36
CDK5: cyclin-dependent kinase 5
ChAT: choline acetyltransferase
CNS: central nervous system
CO: carbon monoxide
CSF: cerebrospinal fluid
Cu: copper
Dkk-1: Dickkopf WNT Signalling Pathway Inhibitor 1
DOHaD: Developmental Origin of Health and Disease
DomA: 3,4-dihydroxymandelic acid
Dvl: Dishevelled
ECF: extracellular fluid
ER: endoplasmic reticulum
ERK: extracellular signal-regulated kinase
ETC: electron transport chain
ETS: electron transport system
FAD: familial AD
Fe: iron
Fz: Frizzled
GABA: γ- aminobutyric acid
GSH-Px: glutathione peroxidase
GSK-3β: Glycogen synthase kinase 3 beta
GSS: glutathione synthetase
H_2_O_2_: hydrogen peroxide
HCP: Hexachlorophene
Hg: mercury
HNE: hyroxynonenal
iGluRs: Ionotropic glutamate receptors
IL- 1β: interleukin - 1β
IL-1: interleukin-1
IL-6: interleukin - 6
IL-12: interleukin-12
IL-18: interleukin -18
IL-23: interleukin-23
IREs: Iron responsive elements
KA: Kainic acid
KARs: Kainate receptors
LDLR: low density lipoprotein receptor
LPS: lipopolysaccharide
LTD: long-term depression
LTP: long-term potentiation
MAPK: mitogen-activated protein kinase
MDA: malonaldehyde
MeHg: methylmercury
mGluRs: metabotropic glutamate receptors
miRNA: micro RNA
Mn: manganese
MnSOD: manganese superoxide dismutase
MPP+: 1-methyl-4-phenylpyridinium
mTOR: mammalian target of rapamycin
Nano-CuO: copper nanoparticles
NEP: neprilysin
NFTs: neurofibrillary tangles
NF-κB: nuclear factor-κB
Ni: nickel
NMDA: N-methyl-D-aspartate
NMDAR: N-methyl-D-aspartate receptor
NO: nitric oxide
NO_2_: nitrogen dioxide
O_3_: ozone
PAHs: polycyclic aromatic hydrocarbons
Pb: lead
PCBs: polychlorinated biphenyls
PCDDs: polychlorinated dibenzo-p-dioxins
PCDFs: polychlorinated dibenzofurans
PGE2: prostaglandin E2
PI3k-Akt: phosphatidylinositol-3-kinase and protein kinase B
PKC: protein kinase C
PM: particulate matter
PP2A: protein phosphate 2 A
PPAR: peroxisome proliferator-activated receptor
PQ: paraquat
PSEN1: presenilin-1
PSEN2: presenilin-2
RACK-1: receptor activated c kinase-1
RNS: reactive nitrogen species
ROS: reactive oxygen species
SAD: sporadic AD
sAβPPα: soluble amyloid-β precursor protein α
Sb: antimony
SiNPs: silica nanoparticles
SO_2_: sulphur dioxide
SOD: superoxide dismutase
SPs: senile plaques
TCC: triclocarban
TCDD: 2,3,7,8-tetrachlorodibenzo-p-dioxin
TCS: triclosan
TFEB: transcription factor EB
TiO_2_NPs: titanium dioxide nanoparticles
TLR: toll like receptor
TMT: 2,4,6-Trimercapto-1,3,5-triazine
TNF-α: tumour necrosis factor-α
Wnt: wingless and Int.
Zn: zinc
